# Supplementary material for: Polyketide synthase gene clusters in the ericoid mycorrhizal fungus Oidiodendron maius: genome-wide prediction, expression profiling, and functional characterization of knockout mutants
Source: Front Plant Sci. 2026 Sep 16;17:1924430. doi: 10.3389/fpls.2026.1924430 (PMC13623903; doi:10.3389/fpls.2026.1924430)
Supplement: Supplementary file 1 [file SupplementaryFile1.zip › Supplementary Materials/Supplementary Folder 1.zip!/Oidma1_AssemblyScaffolds_Repeatmasked/knownclusterblast/cluster39/jgi.p_Oidma1_170889_mibig_hits.html]

| MIBiG Protein | Description | MIBiG Cluster | MiBiG Product | % ID | % Coverage | BLAST Score | E-value |
| --- | --- | --- | --- | --- | --- | --- | --- |
| AMM63173.1 | AniK | BGC0001371 | Echinocandin\_B\_biosynthetic\_gene\_cluster | 26 | 77.1276595745 | 95 | 1.6e-19 |
| AFT91382.1 | EcdK | BGC0000338 | Echinocandin\_B\_biosynthetic\_gene\_cluster | 25 | 77.1276595745 | 89 | 1.2e-17 |
| AKA59462.1 | 2OG\_Fe(II)\_oxygenase | BGC0001203 | Clarexpoxcin\_biosynthetic\_gene\_cluster | 31 | 33.7765957447 | 82 | 1.1e-15 |
| CAM59607.1 | putative\_dioxygenase | BGC0000297 | Aeruginoside\_biosynthetic\_gene\_cluster | 28 | 56.914893617 | 81 | 1.9e-15 |
| EPE34350.1 | Clavaminate\_synthase-like\_protein | BGC0001035 | Pneumocandin\_biosynthetic\_gene\_cluster | 24 | 78.1914893617 | 77 | 3.5e-14 |
